# Network Pharmacology Integrated Molecular Docking Reveals the Mechanism of Anisodamine Hydrobromide Injection against Novel Coronavirus Pneumonia

**DOI:** 10.1155/2020/5818107

**Published:** 2020-08-05

**Authors:** Jinsong Su, Zixuan Liu, Chuan Liu, Xuanhao Li, Yi Wang, Jing Zhao, Qingjiang Wu, Shichao Zheng, Yi Zhang

**Affiliations:** ^1^Ethnic Medicine Academic Heritage Innovation Research Center, Chengdu University of Traditional Chinese Medicine, Chengdu 611137, China; ^2^Institute of Interdisciplinary Integrative Medicine Research, Shanghai University of Traditional Chinese Medicine, Shanghai 200000, China; ^3^Chengdu First Pharmaceutical Co., Ltd., Chengdu 611137, China; ^4^Medical Information Engineering College, Chengdu University of Traditional Chinese Medicine, Chengdu 611137, China

## Abstract

**Background:**

The Coronavirus Disease 2019 (COVID-19) outbreak in Wuhan, China, was caused by severe acute respiratory syndrome coronavirus 2 (SARS-CoV-2). Anisodamine hydrobromide injection (AHI), the main ingredient of which is anisodamine, is a listed drug for improving microcirculation in China. Anisodamine can improve the condition of patients with COVID-19.

**Materials and Methods:**

Protein-protein interactions obtained from the String databases were used to construct the protein interaction network (PIN) of AHI using Cytoscape. The crucial targets of AHI PIN were screened by calculating three topological parameters. Gene ontology and pathway enrichment analyses were performed. The intersection between the AHI component proteins and angiotensin-converting enzyme 2 (ACE2) coexpression proteins was analyzed. We further investigated our predictions of crucial targets by performing molecular docking studies with anisodamine.

**Results:**

The PIN of AHI, including 172 nodes and 1454 interactions, was constructed. A total of 54 crucial targets were obtained based on topological feature calculations. The results of Gene Ontology showed that AHI could regulate cell death, cytokine-mediated signaling pathways, and immune system processes. KEGG disease pathways were mainly enriched in viral infections, cancer, and immune system diseases. Between AHI targets and ACE2 coexpression proteins, 26 common proteins were obtained. The results of molecular docking showed that anisodamine bound well to all the crucial targets.

**Conclusion:**

The network pharmacological strategy integrated molecular docking to explore the mechanism of action of AHI against COVID-19. It provides protein targets associated with COVID-19 that may be further tested as therapeutic targets of anisodamine.

## 1. Background

COVID-19 was first reported in late December 2019 in Wuhan, China and has rapidly spread to more than a dozen of countries, including the United States, with thousands of infected individuals and hundreds of deaths within a month [1]. As of today (25 Feb 2020), 77,785 cases have been identified in China, and 25 cases have been cumulatively reported in Taiwan, China. It is of great importance to search for fast and effective therapeutic drugs for COVID-19. Drug reutilization is a common strategy in the search for antiviral treatment. It has been reported that some approved drugs are beneficial to patients. Chinese patent medicine, in particular, plays a key role in the treatment of viral diseases in China [2]. According to the theoretical foundation of plague, it is convinced that Chinese medicine is an effective treatment for COVID-19 [3].

**Figure 1:**
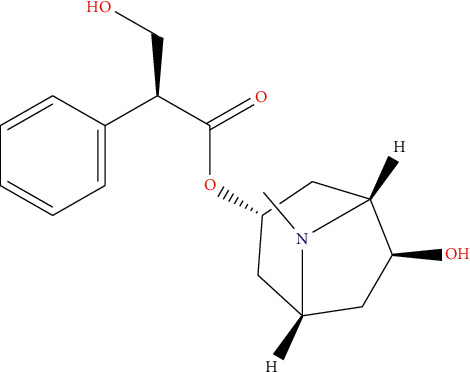
Chemical structure of anisodamine.

AHI, an anti-inflammatory agent, can relieve smooth muscle spasm, inhibit gland secretion and improve pulmonary microcirculation, and it is used clinically to treat pneumonia in China and has been reported to improve COVID-19 [4–6]. The main component of AHI is anisodamine (Figure 1). Anisodamine a tropane alkaloid extracted from the roots of *Anisodus tanguticus* (Maxim.) Pascher. is an acetylcholine receptor blocker. By blocking the muscarinic acetylcholine receptor, anisodamine can reduce glandular secretion, inhibit the hyperexcitability of cholinergic nerves, eliminate muscarinic symptoms, relax the spasm of smooth muscle, and accelerate the heartbeat etc. [7]. It has been used for many diseases, such as respiratory diseases, various circulatory disorders, septic shock, gastric ulcers, migraine, gastrointestinal colic, acute glomerular nephritis, eclampsia, rheumatoid arthritis, obstructive jaundice, opiate addiction, organophosphorus poisoning, and snake bite, as well as in radiation damage protection, due to its ability to improve microcirculation [8, 9]. Anisodamine had also been used to treat patients of severe acute respiratory syndrome (SARS) with hypoxemia in 2003 [10]. Severe acute respiratory syndrome coronavirus 2 (SARS-CoV-2) can cause pulmonary inflammatory reactions in patients, leading to pulmonary microcirculation disorder; therefore patients could have symptoms of dyspnea and hypoxia. AHI can improve symptoms of COVID-19 by regulating pulmonary microcirculation. In this study, we explored the mechanism through which AHI improves COVID-19, as it is of great importance to provide a possible therapeutic schedule for COVID-19.

Network pharmacology is considered a promising approach for a cost-effective drug development and it has been widely used to identify the active ingredients of some traditional Chinese medicine and their mechanisms of actions [11, 12]. It has transformed the research approach of “one target, one drug” into a “network target, multicomponent” strategy. Taking anisodamine as an example, a targeting method of natural products based on the PubChem database has been used. The targeting method is rapid and could provide a relatively accurate result without the support of high-performance computing [13]. Based on the principle of structural biology, molecular docking can be applied to perform virtual drug screening via a computer-assisted drug molecular biological design. Hence, molecular docking is an effective way to find and identify drug targets by filtering the docking energy and space matching between molecules and targets [14]. The integration of in silico and experimental results predicted that Qingfei Paidu Decoction could treat pneumonia in COVID-19 patients and Ma Xing Shi Gan Decoction plays an important role in this [15, 16]. Therefore, the integration of molecular docking and network pharmacology is helpful in accelerating the progress in target discovery and experimental verification. In this study, a network pharmacological strategy integrated with molecular docking was used to explore the mechanism through which AHI improves COVID-19, so as to provide scientific evidence for clinical medication.

## 2. Materials and Methods

### 2.1. Compound Target Prediction

The related targets of anisodamine were extracted from PubChem using the key word “anisodamine.” Next, the component targets were imported to the UniProt database (http://www.UniProt.org/) to obtain the UniProt ID.

### 2.2. Protein Interaction Network (PIN) Construction and Topological Feature Analysis

Using String (https://stringdb.org/, ver. 11.0), the revised targets were used to obtain associated protein-protein interactions for the construction of the PIN of AHI. We limited the species to “*Homo sapiens*” and set the minimum interaction threshold to 0.7. The network construction was visualized using Cytoscape (version 3.7.1). Next, three topological parameters including “Degree,” “Betweenness Centrality,” and “Closeness Centrality” were calculated by Network Analyzer. Just the nodes with “Degree,” “Betweenness Centrality,” and “Closeness Centrality” larger than the corresponding median values were recognized as crucial targets of AHI against COVID-19.

### 2.3. Gene Ontology (GO) and Kyoto Encyclopedia of Genes and Genomes (KEGG) Pathway Enrichment Analyses

The crucial targets were used for GO and KEGG pathway analyses using the String database with the screening criteria of FDR ≤1 × 10^−6^. By using the count score, we selected the top 20 GOs for presentation. The pathway terms with FDR ≤1 × 10^−6^ were mapped to the KEGG human diseases in order to obtain the disease pathway.

### 2.4. Intersection Analysis of the AHI Targets and ACE2 Coexpression Proteins

ACE2 is the SARS-CoV receptor and has been linked to SARS-CoV-2 infection. ACE2 coexpressed proteins were obtained from colonic epithelial cells in the study conducted by Wang et al. [17]. Hence, the intersection calculation between the AHI targets and ACE2 coexpression proteins was performed to obtain the common protein to explore the moderation effect of AHI on ACE2 coexpression proteins.

### 2.5. Molecular Docking between Target and Compound

The crystal structures of candidate protein targets of anisodamine were downloaded from the RCSB Protein Data Bank (http://www.rcsb.org/). Furthermore, modifications including ligands and water removal, hydrogen subjoining, optimization, and patch of amino acid were conducted with Maestro software (Version 11.1.011), while the ionization parameters remained unchanged. The scale factors and partial charge cutoff values of the van der Waals radius scaling 0.25 1 Å were applied to generate grids at the active sites. ChemBioOffice2014 was used to create 3D chemical structures and minimize their energy. The results were saved in MOL.2 format. The docking score between the compound and the target protein was presented as kcal/mol and used as the evaluation criteria to further screen out potential active components. Targets with docking scores >4.25 were considered meaningful [18].

## 3. Results

### 3.1. Information on Compound Targets

By searching PubMed, a total of 88 targets of anisodamine were obtained, and the target information is shown in Table S1.

### 3.2. PIN Construction and Topological Analysis

**Figure 2:**
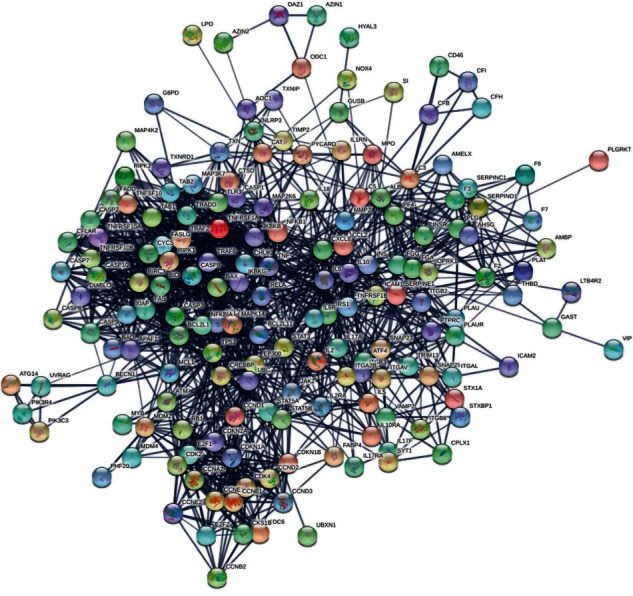
Protein-protein interaction network (PIN) of AHI consists of 172 nodes and 1454 interactions.

**Figure 3:**
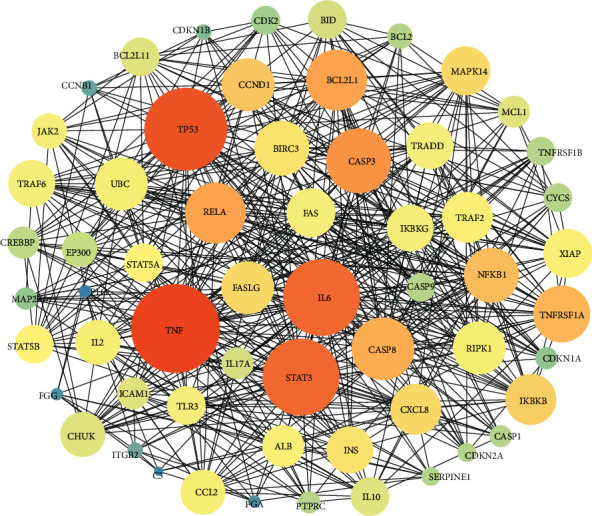
Crucial target of PIN of AHI. The node size is proportional to the node degree, the color is darker, and the node betweenness is larger.

The PIN of AHI consisted of 172 nodes and 1454 interactions, as shown in Figure 2. Three topological features—“Degree,” “Betweenness,” and “Closeness”—were calculated for each node in the network to screen the crucial hubs with topological importance (Supplementary Data Table S2). The median values of Degree, Betweenness, and Closeness were 16, 0.00285052, and 0.41707317, respectively. Furthermore, we determined that nodes with “Degree” >16, “Node betweenness” >0.00285052, and “Closeness” >0.41707317 were the crucial targets. As a result, 54 crucial targets were obtained (Figure 3). As shown in Figure 3, the top five degrees among the crucial targets were TP53, TNF, IL6, CASP3, and CASP8, and their degrees were 60, 59, 53, 46, and 43, respectively. TP53, TNF, and IL6 are closely related to the regulation of inflammation [19–21], while CASP3 and CASP8 play an important role in the apoptosis process [22, 23].

### 3.3. Gene Ontology and KEGG Pathway Analysis

**Figure 4:**
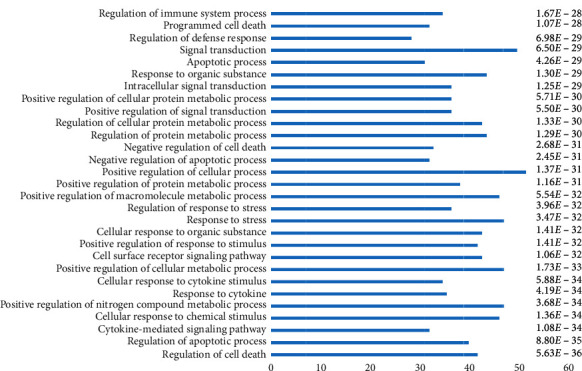
Top 30 biological processes of the crucial targets of AHI.

To elucidate the mechanisms of AHI against COVID-19, we conducted GO biological processes and pathway enrichment analysis for crucial targets. The top 30 biological processes involved in AHI against COVID-19 are shown in Figure 4. The biological processes mainly includes the regulation of cell death, cytokine-mediated signaling pathway, cellular response to chemical stimulus, positive regulation of nitrogen compound metabolic process, response to cytokine, cellular response to cytokine stimulus, positive regulation of cellular metabolic process, cell surface receptor signaling pathway, regulation of immune system process, etc.

**Figure 5:**
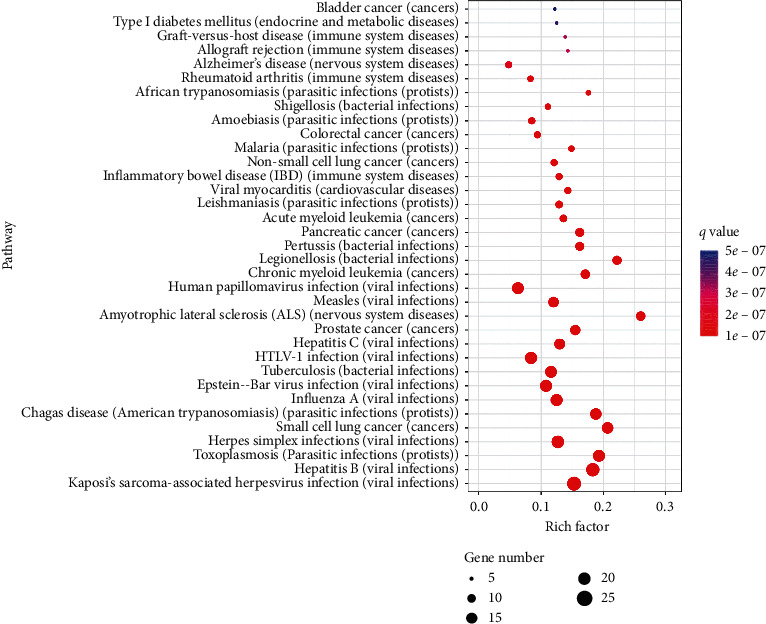
KEGG analysis of disease pathways regulated by AHI's crucial targets.

KEGG pathway enrichment analysis was executed based on the String database (as shown in Figure 5). The results showed that the diseases mainly involved are viral infections, cancer, and immune system diseases, which are associated with inflammation. They include diseases such as influenza A, HTLV-I infection, small cell lung cancer, inflammatory bowel disease, human papillomavirus, hepatitis B, tuberculosis, hepatitis C, allograft rejection, rheumatoid arthritis, Shigellosis, colorectal cancer, amoebiasis, malaria, viral myocarditis, leishmaniasis, acute myeloid leukemia, pancreatic cancer, and pertussis.

### 3.4. Intersection Analysis of the AHI Targets and ACE2 Coexpression Proteins

A total of 5556 proteins coexpressed with ACE2 were obtained. By calculating the intersection between the AHI component proteins and ACE2 coexpression proteins, 26 common proteins were obtained (shown in Table 1). Among them, BCL2L1, CASP1, CASP3, CASP8, IL17A, and STAT3 were crucial targets of AHI.

### 3.5. Molecular Docking

**Figure 6:**
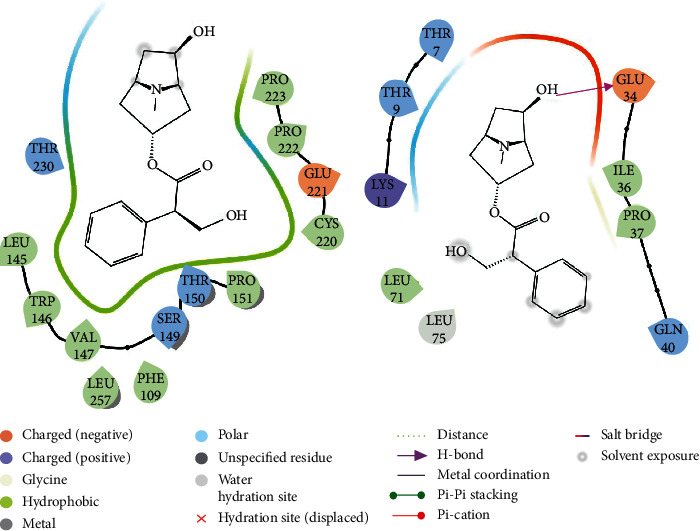
Schematic representation of the molecular docking between TP53, UBC target proteins, and anisodamine (2D).

**Figure 7:**
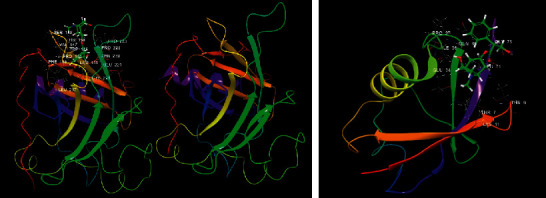
Schematic representation of the molecular docking between TP53, UBC target proteins, and anisodamine. Major amino acids are depicted as thin tubes, and atoms are colored gray (carbon), white (hydrogen), blue (nitrogen), and red (oxygen). Abbreviations and serial numbers of the amino acids are included. Thick tubes represent ligands. The carbon atoms of the ligand are in green. Yellow dotted lines represent hydrogen bonding (3D).

**Figure 8:**
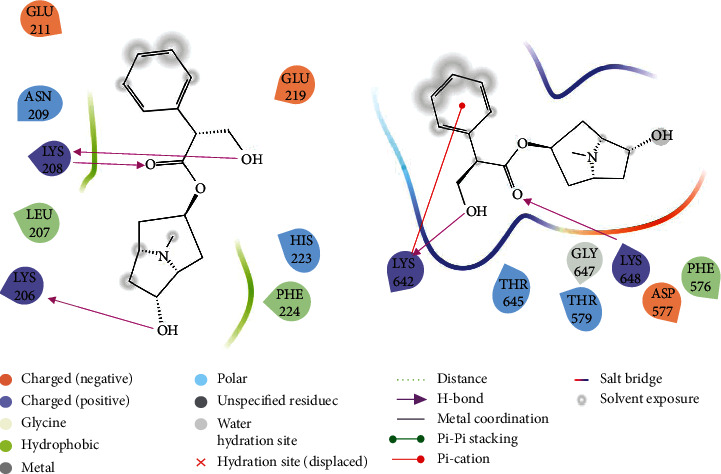
Schematic representation of the molecular docking between XIAP, RIPK1 target proteins, and anisodamine (2D).

**Figure 9:**
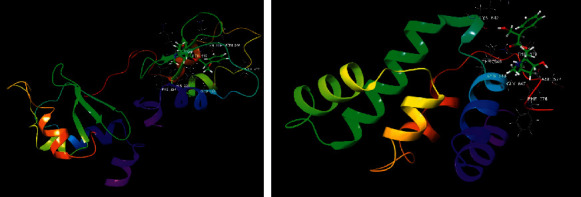
Schematic representation of the molecular docking between XIAP, RIPK1 target proteins, and anisodamine. Major amino acids are depicted as thin tubes, and atoms are colored gray (carbon), white (hydrogen), blue (nitrogen), and red (oxygen). Abbreviations and serial numbers of the amino acids are included. Thick tubes represent ligands. The carbon atoms of the ligand are in green. Yellow dotted lines represent hydrogen bonding (3D).

The crucial targets of TP53, UBC, XIAP, and RIPK1 were chosen to further study the accuracy of prediction results by calculating the interaction with anisodamine using molecular docking. The results of molecular docking indicated that all anisodamines entered the active pocket of the enzyme; for example, anisodamine entered the active pocket of TP53. Interestingly, although there was no combination of hydrogen and aromatic bonds with the compound (Figures 6 and 7), anisodamine combined with an active pocket through van der Waals forces, and the docking scores reached 5.636. Anisodamine bound to the active pockets of UBC and one hydroxyl interacted with GLU34 to form a hydrogen bond, as shown in Figure 6. However, anisodamine bound to the active pockets of XIAP and two hydroxyl groups interacted with LYS206 and LYS208 to form hydrogen bonds, the carbonyl also interacted with LYS208 to form a hydrogen bond (Figures 8 and 9). Similarly, anisodamine was linked to the active pocket of RIPK1, and the carbonyl interacted with LYS648 to form a hydrogen bond. Besides, the hydroxyl interacted with LYS642 to form a hydrogen bond, and the aromatic ring interacted with LYS642 to form aromatic hydrogen bonds (Figure 8).

## 4. Discussion

There is a worldwide concern about COVID-19 as a global public health threat as SARS-CoV-2 causes severe acute respiratory syndrome [24, 25], and this has caused the death of many patients. In addition, it has been reported that ACE2 is the main host cell receptor of SARS-CoV-2 and plays a key role in the viral infection [26]. ACE2 could cause various inflammation, it has been proved that AHI can improve microcirculation in the body, and it has been used to treat pneumonia, septic shock, and other diseases [6, 27, 28]. Some studies indicated that AHI combined with azithromycin sequential therapy in the treatment of mycoplasma pneumonia infection could improve the clinical curative effect, decrease the serum inflammatory cytokines, and increase immunological markers [29, 30]. AHI can improve COVID-19 by eliminating inflammation and promoting microcirculation. This study used network pharmacology and molecular docking technology to predict the potential targets of anisodamine as the main active component of AHI in the treatment of COVID-19.

ACE2 is a key negative regulatory factor for the severity of lung edema and acute lung failure; therefore, the mediation of ACE2 downregulation could contribute to the severity of lung pathologies [31]. We screened out some coexpressed targets of anisodamine and ACE2, including IL17A, STAT3, AOC1, BCL2L1, CASP1, CASP3, CASP8, CTSD, DNAH8, ICAM2, IL18, IL5, ITGAV, KCNA3, MPO, MTMR11, NOX4, ODC1, PLAUR, PLGRKT, RSAD2, SERPINC1, TIMP2, TXN, TXNIP, and VIP. For example, IL17A is a proinflammatory cytokine that critically regulates host defense against multiple pathogens. You et al. used a Pts4^d/d^-driven mouse model to confirm that IL17A could regulate tumor latency and metastasis in lung adenocarcinoma and squamous cell carcinoma [32]. Evidence indicated that treatment with anisodamine could markedly reduce the expression of IL17A and IL17F in rats [33]. A study also showed that the levels of MMP2 and TIMP2 mRNA and the protein of MMP2 in livers could be significantly reduced in the anisodamine preventive group and therapeutic groups [34]. STAT3 signaling is linked to multiple pathways and influences both immunity and inflammatory injury. In *E. coli* pneumonia, STAT3 can promote neutrophil recruitment and limit both infection and lung injury [35]. Choi et al. also reported the antibacterial function of STAT3 in the lung epithelium, which was mediated by the induction of Reg3*γ* [36]. BCL2L1 is an inflammation-protective gene that can regulate apoptosis. Previous studies confirmed that BCL2L1 negatively correlated with the survival of lung cancer patients. Zhang et al. used cell experiments to demonstrate that let-7a-5p could directly target BCL2L1, inhibit BCL2L1 expression, and suppress lung cancer cell proliferation, migration, and invasion [37]. In another study, the antiapoptotic effect of A1AT in alveolar endothelial cells via direct CASP3 inhibition was demonstrated. This result represents a novel antiapoptotic mechanism relevant to disease processes characterized by inflammation, such as pulmonary emphysema [38]. Yuan et al. showed that anisodamine treatment could reduce expression of ER stress markers IRE-1*α*, CHOP, and ATF4, TXNIP, and NLRP3, as well as ACS, CASP1, IL1*α*, IL1*β*, and IL18 [39]. Evidently, anisodamine can indirectly limit the expression of ACE2 by acting on coexpressed targets.

In this study, the biological processes mainly include the regulation of cell death, cytokine-mediated signaling pathway, immune system process, etc. Apoptosis and autophagy are common pathways that regulate cell death and have been extensively studied. It is through the regulation of cell death that the body regulates normal functions. For example, some studies demonstrated that targeting autophagy may be used in the future for the treatment of lung cancer because the disruption of autophagy via the inhibition of Atg5 and Beclin 1 may promote cisplatin-induced apoptotic cell death in A549 human lung cancer cells [40]. The immune depression syndrome caused by spinal cord injury induces pneumonia; however, Tiphaine et al. applied a new model to confirm that the blockade of the negative costimulatory molecule PD-1 decreases the rate of secondary bacterial pneumonia through an increase in IFN-*γ* production by NKT cells [41]. Zou et al. showed that Morf4l1 may be a potential target for the treatment of severe pulmonary infection because, in mice infected with *P. aeruginosa*, the overexpression of Morf4l1 resulted in lung epithelial cell death, and its depletion restored the vitality of cells [42]. In lung cancer, Baumert et al. used RNA-Seq, mass spectrometry, and linked them with functional cell culture to identify the function of KMT9. The results showed that the depletion of KMT9 inhibited lung cancer cell proliferation by inducing nonapoptotic cell death [43]. Evidence from several clinical studies indicates that a complex network of inflammatory cytokines and chemokines play a major role in mediating, amplifying, and perpetuating the lung injury process. Goodman et al. found that the lung-protective ventilatory strategy reduced mortality by 22%, which is related to the concentration of proinflammatory cytokines released into the injured lung airspace [44]. In addition, the cytokine-mediated signaling pathway has potential significance in the treatment of pneumonia. Menna et al. examined if Fc*γ*RIIb could control the balance between defense and septic shock in response to *S. pneumoniae*. Moreover, Fc*γ*RIIb-deficient mice showed increased phagocytosis of pneumococci by macrophages *in vitro* and increased bacterial clearance and survival *in vivo* [45]. The cytokines interleukin-17 and interleukin-22 are produced by the T-helper-17-cell subset and are regarded as crucial regulators of antimicrobial peptide production in the gut and lungs. This suggests that T-cell lineage and its cytokines play important roles in skin and mucosal immunity [46]. Komaki et al. used a new bronchoscopic microsampling technique and found that xanthine oxidase (XO) activity is increased in chronic obstructive pulmonary disease (COPD), possibly due to its gene upregulation by proinflammatory cytokines. Consequently, cytokine-XO production pathways might play an important role in the development of inflammation in COPD [47]. The immune system process is also closely linked to the treatment of pneumonia. Roux et al. used a rat model to indicate that *C. albicans* airway colonization could promote the development of bacterial pneumonia; however, this effect was inhibited *in vitro* because the inhibition of AM phagocytosis in relation to a Th1-Th17 immune response was triggered by fungal colonization [48]. Alveolar macrophages (AM) are believed to be of central importance to the initial host response to pulmonary cryptococcal infection because they constitute the major resident phagocytic cells in the lungs. The study suggested that the differences in susceptibility to pulmonary cryptococcal infection in rats and mice may result from basic differences in AM [49]. The immune system plays an important role in lung function. Alekseevskikh et al. studied 74 children aged from 1 month to 1 year who died from acute pneumonia and recommended that patients should undergo early immunomodulatory treatment [50]. The immune system process was also closely related to pneumonia. In 76 patients with acute pneumonia, the absolute and relative numbers of T- and B-lymphocytes were determined. Bykova et al. observed that there was a sharp decrease in the number and function of T- and B-lymphocytes in croupous pneumonia and in the prolonged course of local pneumonia [51]. Therefore, we found that these signaling pathways are closely related to pneumonia and inflammation and that AHI may improve COVID-19 through the regulation of signaling pathways.

The viral infections, bacterial infections, and parasitic infections were analyzed via KEGG enrichment analysis. Novel coronaviruses have been isolated from human airway epithelial cells, and SARS-CoV-2 is the seventh member of the family of coronaviruses [52]. Currently, remdesivir and chloroquine are recognized as promising antiviral drugs. A recent study indicated that remdesivir is an adenosine analog that is incorporated into nascent viral RNA chains and results in premature termination. However, chloroquine blocks viral infection by increasing endosomal pH required for virus/cell fusion and interferes with the glycosylation of cellular receptors of SARS-CoV [53]. Both remdesivir and chloroquine treat COVID-19 by blocking the synthesis of the virus. Therefore, AHI may indirectly or directly regulate various inflammatory pathways, immune pathways, and related targets to prevent viral infections.

As Table 2 shows, the higher the docking score, the better the docking effect. According to all the docking results, anisodamine bound well to all targets, and the docking scores of TP53 and XIAP were 5.636 and 4.411, respectively. The results of this study indicate that AHI is a potential therapeutic agent for COVID-19.

## 5. Conclusion

In this study, in an attempt to provide a possible therapeutic schedule for COVID-19, a network pharmacology method and molecular docking technology were integrated to predict the mechanism through which AHI improves COVID-19. AHI may regulate cell death, cytokine-mediated signaling pathway, and immune system process to combat viral infections, cancer, and immune system diseases. Through the intersection analysis between the AHI targets and ACE2 coexpression proteins, we observed that AHI may indirectly limit the expression of ACE2 by acting on coexpressed targets. Therefore, AHI may be a potential drug for the treatment of COVID-19. Nevertheless, further experiments are needed to support our findings. The present study revealed the mechanisms through which AHI improves COVID-19. It provides an important basis for further study on the mechanisms of AHI and the optimization of experimental designs for more reliable experimental results.

## Figures and Tables

**Table 1:** Common targets of AHI component proteins shared with ACE2 coexpression proteins.

Common proteins	UniProt ID
AOC1	P19801
BCL2L1^*∗*^	Q07817
CASP1^*∗*^	P29466
CASP3^*∗*^	P42574
CASP8^*∗*^	O15519
CTSD	P07339
DNAH8	Q96JB1
ICAM2	P35330
IL17A^*∗*^	Q16552
IL18	Q14116
IL5	P05113
ITGAV	P06756
KCNA3	P22001
MPO	P05164
MTMR11	A4FU01
NOX4	Q9NPH5
ODC1	P11926
PLAUR	Q03405
PLGRKT	Q9HBL7
RSAD2	Q8WXG1
SERPINC1	P01008
STAT3^*∗*^	P40763
TIMP2	P16035
TXN	P10599
TXNIP	Q9H3M7
VIP	P01282

^*∗*^Crucial targets.

**Table 2:** Correlation score of molecular docking.

Target	Docking score	Glide gscore	Glide evdw	Glide ecoul	Glide emodel	Glide energy
TP53	−5.636	−5.636	−27.115	−1.124	−34.815	−28.239
UBC	−3.512	−3.512	−13.346	−13.057	−31.77	−26.402
XIAP	−4.411	−4.411	−21.804	−7.97	−36.497	−29.774
RIPK1	−2.826	−2.826	−17.448	−6.351	−25.911	−23.799

## Data Availability

The data used to support the findings of this study are available from the corresponding author upon request.

## Supplementary Materials

Supplementary Materials Table S1: the detailed information of targets of anisodamine. Table S2: the information of crucial targets of AHI's PIN.
