# Supplementary material for: Network Pharmacology Integrated Molecular Docking Reveals the Mechanism of Anisodamine Hydrobromide Injection against Novel Coronavirus Pneumonia
Source: Evid Based Complement Alternat Med. 2020 Aug 5;2020:5818107. doi: 10.1155/2020/5818107 (PMC7411467; doi:10.1155/2020/5818107)
Supplement: Supplementary Materials — Table S1: the detailed information of targets of anisodamine. Table S2: the information of crucial targets of AHI's PIN. [file 5818107.f1.zip › TableS1.docx]

**Table S1.** the detail information of targets of anisodamine

| Target | Uniprot ID | Target | Uniprot ID |
| --- | --- | --- | --- |
| ABCC6 | O95255 | ITGAV | P06756 |
| ACPP | P15309 | JAK2 | O60674 |
| ADH1A | P07327 | KCNA3 | P22001 |
| ALB | P02768 | KCNMB4 | Q86W47 |
| AMBP | P02760 | LPO | P22079 |
| AMELX | Q99217 | LTB4R2 | Q9NPC1 |
| AOC1 | P19801 | MAP4K2 | Q12851 |
| ATF4 | P18848 | MAPK14 | Q16539 |
| AZIN2 | Q96A70 | MMP2 | P08253 |
| BCL2 | P10415 | MOCOS | Q96EN8 |
| BCL2L1 | Q07817 | MPO | P05164 |
| C3 | P01024 | MTMR11 | A4FU01 |
| C5 | P01031 | MYB | P10242 |
| CASP1 | P29466 | NFKB1 | P19838 |
| CASP3 | P42574 | NLRP3 | Q96P20 |
| CASP8 | Q14790 | NOX4 | Q9NPH5 |
| CAT | P04040 | ODC1 | P11926 |
| CCL2 | P13500 | OPRK1 | P41145 |
| CCS | O14618 | PF4 | P02776 |
| CKMT1B | P12532 | PGA5 | P0DJD9 |
| CTSD | P07339 | PHF20 | Q9BVI0 |
| CXCL8 | P10145 | PIK3AP1 | Q6ZUJ8 |
| DIABLO | Q9NR28 | PLAUR | Q03405 |
| DNAH8 | Q96JB1 | PLG | P00747 |
| DUSP11 | O75319 | PLGRKT | Q9HBL7 |
| F3 | P13726 | PTPRC | P08575 |
| FABP4 | P15090 | PYCARD | Q9ULZ3 |
| G6PD | P11413 | RB1 | P06400 |
| GAST | P01350 | RIPK3 | Q9Y572 |
| GJA1 | P17302 | RSAD2 | Q8WXG1 |
| GUSB | P08236 | SERPINC1 | P01008 |
| HYAL3 | O43820 | SERPINE1 | P05121 |
| ICAM1 | P13597 | SI | P14410 |
| ICAM2 | P13598 | STAT3 | P40763 |
| IKBKB | O14920 | SYT1 | P21579 |
| IL10 | P22301 | TIMP2 | P16035 |
| IL17A | Q16552 | TNF | P01375 |
| IL17F | Q96PD4 | TNFRSF1B | P20333 |
| IL18 | Q14116 | TRIM13 | O60858 |
| IL1RN | P18510 | TXN | P10599 |
| IL2 | P60568 | TXNIP | Q9H3M7 |
| IL5 | P05113 | UBXN1 | Q04323 |
| IL6 | P05231 | VIP | P01282 |
| INS | P01308 | WBP2NL | Q6ICG8 |
