# Supplementary material for: Network Pharmacology Integrated Molecular Docking Reveals the Mechanism of Anisodamine Hydrobromide Injection against Novel Coronavirus Pneumonia
Source: Evid Based Complement Alternat Med. 2020 Aug 5;2020:5818107. doi: 10.1155/2020/5818107 (PMC7411467; doi:10.1155/2020/5818107)
Supplement: Supplementary Materials — Table S1: the detailed information of targets of anisodamine. Table S2: the information of crucial targets of AHI's PIN. [file 5818107.f1.zip › TableS2.docx]

**Table S2.** the information of crucial target of AHI's PIN

| Crucial targets | Degree | Betweenness Centrality | Closeness Centrality |
| --- | --- | --- | --- |
| TP53 | 60 | 0.10571566 | 0.56810631 |
| TNF | 59 | 0.0710955 | 0.56810631 |
| IL6 | 53 | 0.10616925 | 0.56435644 |
| CASP3 | 46 | 0.04262644 | 0.52293578 |
| CASP8 | 44 | 0.02114677 | 0.48305085 |
| STAT3 | 43 | 0.04257305 | 0.53605016 |
| BCL2L1 | 40 | 0.04058026 | 0.51818182 |
| UBC | 39 | 0.0611924 | 0.47765363 |
| XIAP | 36 | 0.00816563 | 0.46216216 |
| RIPK1 | 36 | 0.00531574 | 0.45844504 |
| CCND1 | 36 | 0.02158945 | 0.48857143 |
| TRAF2 | 36 | 0.00587163 | 0.46216216 |
| TNFRSF1A | 36 | 0.00938218 | 0.48717949 |
| INS | 35 | 0.12755121 | 0.52777778 |
| BIRC3 | 35 | 0.00626037 | 0.45844504 |
| RELA | 35 | 0.0121325 | 0.49854227 |
| NFKB1 | 34 | 0.03852595 | 0.5074184 |
| IKBKB | 34 | 0.01695461 | 0.5074184 |
| IKBKG | 33 | 0.01013325 | 0.47107438 |
| TRADD | 32 | 0.00413889 | 0.45478723 |
| FASLG | 31 | 0.005519 | 0.44186047 |
| CHUK | 31 | 0.00823761 | 0.46594005 |
| CXCL8 | 31 | 0.03046492 | 0.50591716 |
| FAS | 29 | 0.00460004 | 0.44415584 |
| ALB | 28 | 0.02622766 | 0.48997135 |
| CDK2 | 27 | 0.00772974 | 0.44186047 |
| EP300 | 27 | 0.01693233 | 0.47237569 |
| BID | 26 | 0.00518114 | 0.44186047 |
| TRAF6 | 26 | 0.00560525 | 0.45 |
| CCL2 | 26 | 0.01228898 | 0.49279539 |
| MAPK14 | 26 | 0.0057039 | 0.47237569 |
| CREBBP | 25 | 0.01386734 | 0.456 |
| CDKN1A | 25 | 0.00594697 | 0.43846154 |
| IL10 | 25 | 0.00610906 | 0.46594005 |
| JAK2 | 24 | 0.01131722 | 0.48033708 |
| CDKN1B | 24 | 0.0123696 | 0.43622449 |
| CASP9 | 24 | 0.00295681 | 0.44186047 |
| IL2 | 24 | 0.00349407 | 0.456 |
| CYCS | 24 | 0.01069645 | 0.46849315 |
| STAT5B | 23 | 0.00940873 | 0.47632312 |
| STAT5A | 23 | 0.00940873 | 0.47632312 |
| TLR3 | 23 | 0.00421842 | 0.46216216 |
| SERPINE1 | 22 | 0.02042182 | 0.48033708 |
| BCL2L11 | 22 | 0.01236047 | 0.45478723 |
| ICAM1 | 22 | 0.01376615 | 0.48169014 |
| FGA | 22 | 0.01195337 | 0.42857143 |
| CDKN2A | 21 | 0.00805912 | 0.45478723 |
| ITGB2 | 21 | 0.02018552 | 0.43846154 |
| C3 | 21 | 0.052009 | 0.42118227 |
| TNFRSF1B | 20 | 0.01281598 | 0.44300518 |
| CCNB1 | 20 | 0.00328812 | 0.42014742 |
| FGG | 20 | 0.00617526 | 0.42643392 |
| PLG | 20 | 0.01868925 | 0.42118227 |
| BCL2 | 20 | 0.0091494 | 0.45118734 |
| MCL1 | 19 | 0.00307998 | 0.456 |
| CASP1 | 19 | 0.00647282 | 0.4535809 |
| IL17A | 18 | 0.00380625 | 0.44300518 |
| MAP2K6 | 17 | 0.0041328 | 0.42431762 |
| PTPRC | 17 | 0.00733623 | 0.45 |
